# Huajuxiaoji Formula Alleviates Phenyl Sulfate-Induced Diabetic Kidney Disease by Inhibiting NLRP3 Inflammasome Activation and Pyroptosis

**DOI:** 10.1155/2024/8772009

**Published:** 2024-07-15

**Authors:** Zeng Zhang, Yueping Bi, Fengzhu Zhou, Duanchun Zhang, Siyu Xu, Xinyi Zhang, Zhaohua Fan, Zheng Yao, Yanming He

**Affiliations:** ^1^ Department of Endocrinology Yueyang Hospital of Integrated Traditional Chinese and Western Medicine Shanghai University of Traditional Chinese Medicine, Shanghai 200437, China; ^2^ Department of Chinese Medicine Yinhang Community Health Service Center of Yangpu District, Shanghai 200438, China

**Keywords:** diabetic kidney disease, Huajuxiaoji formula, NLRP3 inflammasome, pyroptosis

## Abstract

**Background:** One of the most common microvascular complications of diabetes is diabetic kidney disease (DKD). The Huajuxiaoji formula (HJXJ) has shown clinical efficacy for DKD; however, its regulatory mechanisms against DKD remain elusive. We investigated NLRP3 inflammasome and the mechanisms of HJXJ by which HJXJ alleviates DKD.

**Methods:** Phenyl sulfate (PS) was used to establish DKD models. HJXJ was administered to mice through intragastric or made into a pharmaceutical serum for the cell cultures. Biological indicator levels in mouse blood and urine were analyzed, and kidney tissues were used for HE, Masson, and PAS staining. ELISA and western blotting were used to detect inflammatory cytokines and protein levels, respectively. Reactive oxygen species (ROS) production and pyroptosis were evaluated using flow cytometry. Lentiviral vector-mediated overexpression of NLRP3 was performed to determine whether NLRP3 participates in the antipyroptotic effect of HJXJ.

**Results:** HJXJ significantly reduced the severity of the injury and, in a dose-dependent manner, decreased the levels of biological markers including creatinine, blood urea nitrogen, urine protein, and endotoxin, as well as inflammatory cytokines such as interleukin (IL)-1*β*, IL-18, tumor necrosis factor-*α*, and IL-6 in DKD mice. Treatment with HJXJ reversed the downregulation of podocin, nephrin, ZO-1, and occludin and upregulated ROS, NLRP3, Caspase-1 P20, and GSDMD-N induced by PS. Moreover, the upregulation of NLRP3 expression increased the number of cells positive for pyroptosis. HJXJ suppressed pyroptosis and inflammasome activation by inhibiting NLRP3 expression.

**Conclusions:** Generally, HJXJ has the potential to reduce DKD injury and exerts anti-DKD effects by inhibiting the NLRP3-mediated NLRP3 inflammasome activation and pyroptosis *in vitro* and *in vivo.*

## 1. Introduction

The main cause of end-stage renal disease (ESRD) is diabetic kidney disease (DKD), a significant microvascular complication of diabetes [1]. The prevalence of DKD in diabetic patients is 20%–40%, which rapidly increases in many countries [2]. Type 2 DKD affects 30%–50% of community patients and approximately 40% of hospitalized patients from 2009 to 2012 [3, 4]. However, DKD typically has an insidious onset, and the rate of progression to ESRD accelerates significantly once massive proteinuria is reached [5]. Therefore, early diagnosis and treatment are crucial in improving the quality of life for diabetic patients. Exploring potential mechanisms and effective treatments for DKD has become urgent.

Unnecessary or excessive cell death during the progression of kidney disease can deplete essential parenchymal cells and ultimately lead to renal tissue damage [6]. Pyroptosis, a form of inflammation-induced cell death, was initially misidentified as morphological alterations of apoptosis [7]. Similar to apoptosis, pyroptosis can be initiated by caspases, but it is predominantly induced by proinflammatory caspases—1, 4, 5, and 11, and involves in the formation of inflammasomes, GSDM-dependent cell membrane rupture, and the release of inflammation cytokines [8–11]. Pyroptosis has the potential to trigger a potent inflammatory response, which is particularly significant in the context of DKD, and the role of persistent inflammation is essential in understanding the pathophysiology of DKD [12–14]. The chronic low-grade inflammatory environment, along with the production of insulin-resistant cytokines, tends to exacerbate pyroptosis levels, and the inflammation and damage caused by pyroptosis can worsen renal fibrosis, glomerulosclerosis, and tubular injury [15, 16]. Pyroptosis appears to be an important regulatory process in DKD.

Previous studies reported a link between kidney disease and inflammasomes, especially the nucleotide-binding oligomerization domain-like receptor family pyrin domain-containing protein 3 (NLRP3) inflammasome [17]. The NLRP3 inflammasome is a cytosolic protein complex consisting of the adapter, apoptosis-associated speck-like protein containing a caspase recruitment domain (ASC), and inflammatory cysteine proteinase-1 (caspases-1) [6, 18]. Interestingly, the primary trigger for pyroptosis is the NLRP3 inflammasome, which participates in the classical caspase 1-mediated pyroptosis model [19]. NLRP3, an essential protein within the NLRP3 inflammasome, is responsible for capturing danger signals and recruiting downstream molecules to activate the NLRP3 inflammasome [20]. Additionally, NLRP3 is linked to DKD through metabolic stimuli and reactive metabolites, such as reactive oxygen species (ROS) [21]. Studies showed increased NLRP3 mRNA in the kidneys of patients with Type 2 diabetes and DKD [22, 23]. The NLRP3 is involved in multiple pathways in DKD, including nuclear factor E2-related factor 2, ROS/TXNIP, NF-*κ*B, and P2X7/NLRP3 pathways [24, 25]. What is more, the NLRP3 inflammasome can be activated in renal tubules of diabetic patients with tubulointerstitial injury and glomerular endothelial cells and in podocytes of mice with DKD [26, 27]. Therefore, the NLRP3 inflammasome plays a crucial role in inducing renal inflammation response, and small-molecule inhibitors targeting its components may be potential drugs for treating kidney-related diseases.

Traditional Chinese medicine has been reported to reduce blood pressure and lipids and improve proteinuria symptoms [28]. It is also beneficial for DKD treatment due to its ability to target various clinical and pathological manifestations [28, 29]. The Huajuxiaoji formula (HJXJ) is an ancient formula with many names, such as Yiqi Huaju Qingli formula [30] or Yi-Qi-Zeng-Min-Tang [31]. HJXJ positively impacts metabolic diseases, including diabetes, abnormal glucose regulation, metabolic syndrome, hyperlipidemia, central obesity, and hypertension. Moreover, it is clinically effective for DKD and has been used to alleviate insulin resistance. DKD is a clinical syndrome characterized by persistent albuminuria and decreased estimated glomerular filtration rate [32]. HJXJ can attenuate urinary microalbumin in patients with metabolic syndrome and nephropathy [30]. Animal experiments confirmed that it can reduce insulin resistance in rats with Type 2 diabetes, improve glucose and lipid metabolism, and reduce proteinuria in rats with DKD [31]. Additionally, HJXJ effectively regulates the inflammatory response and alleviates renal fibrosis [31]. These studies provide evidence supporting the potential effects of HJXJ for DKD.

The studies of Zuogui-Jiangtang-Yishen decoction [33] and Tangshen Formula [34] suggest that pyroptosis is a potential target of DKD therapy. Because of the significant impact of HJXJ and the key role of NLRP3 inflammasome activation in DKD development, we hypothesized that the therapeutic effects of HJXJ might be exerted by regulating the NLRP3 inflammasome activation and pyroptosis pathway in DKD. To investigate this hypothesis, we used MPC5 cells and C57BL/KsJ mice, which have metabolic disorders similar to Type 2 diabetes in humans. This study may provide a foundation to understand its therapeutic mechanism.

## 2. Materials and Methods

### 2.1. Animal Study

We obtained 8-week-old male diabetic mice (db/db) with a C57BL/KsJ background and C57 mice from Shanghai Ruitaimos Biotechnology Co., Ltd (China). The phenotype of the diabetic mice was similar to that of a previous study [35]. The db/db diabetic mice were randomly divided into four groups, including a PS group (*N* = 10), a PS + 0.9 g/kg HJXJ group (*N* = 10), a PS + 1.8 g/kg HJXJ group (*N* = 10), and a PS + 3.6 g/kg HJXJ group (*N* = 10). The db/db diabetic mice were administered 50 mg/kg/day phenyl sulfate (PS) (Sundia MediTech, Shanghai, China) orally to induce podocyte injury over 6 weeks [36]. Mice in different treatment groups were administered HJXJ orally at varying doses (0.9, 1.8, and 3.6 g/kg/day) along with PS. Mice in the PS group were orally administered an equal volume of saline instead of HJXJ. The C57 mice (*N* = 10) were only administered saline, and they acted as a control group. After 6 weeks of treatment, the mice were humanely euthanized with 150 mg/kg of pentobarbital sodium (i.p.), following which blood and urine samples were obtained for analyzing the kidney, and kidney samples were obtained for pathological diagnosis. The Animal Experimentation Ethics Committee of the Hospital approved this experiment (YYLAC-2023-206).

### 2.2. HJXJ Preparation

The HJXJ formula contains 12 Chinese herbs, including *Astragalus mongholicus* (30 g), *Rhizoma coptis chinensis* (10 g), *Pollen Typhae* (15 g), *Alisma orientalis* (15 g), *Phaseolus radiatus L.* (30 g), *Serissa serissoides* (15 g), *Radix aconiti lateralis* (9 g), and *Centella asiatica* (15 g). After weighing the raw materials mentioned above, they were added to distilled water at a ratio of 1:8 (w/w) and decocted for 1 h. The mixture was then filtered, and the filtrate was collected. The residue was decocted again to ensure thorough extraction. Subsequently, the two batches of extracts were combined, and then, the pooled solution was concentrated using a rotary evaporator to achieve a crude drug (2 kg/L). The concentrated extract was aliquoted into 15 mL centrifuge tubes, and the opening of each filled tube was sealed with parafilm. These tubes were placed in a freeze-dryer for 48 h, after which they were converted into a powder.

### 2.3. Histology

Kidney tissue sections (*n* = 6 per group) were fixed in 4% paraformaldehyde and then embedded in paraffin. The samples were cut into thin sections and dehydrated. The sections were stained with hematoxylin and eosin (H&E; 60524ES60, Yeasen Biotechnology Co., Ltd, Shanghai, China) by immersing them in hematoxylin for 4 min and subsequently in eosin for 1 min. To perform the Masson trichrome staining (MASSON; 60532ES58, Yeasen Biotechnology Co., Ltd), the sections were stained with hematoxylin for 2 min, followed by immersion in acid fuchsin for 1 min. Next, differentiation was performed for 8 min using the phosphomolybdate color separation solution, and counterstaining was performed for 5 min with aniline blue. To perform periodic acid–Schiff staining (PAS; G1281, Solarbio, Beijing, China), the sections were oxidized in PAS oxidant for 6 min, after which they were stained with the Schiff reagent for 15 min. The nuclei were counterstained with hematoxylin for another 2 min. The staining results were observed under a microscope (CKX3-SLP, OLYMPUS, Japan).

### 2.4. Urine Albumin and Endotoxin Assay Measurement

Assay kits were used to detect creatinine (CRE; BC4910, Solarbio), blood urea nitrogen (BUN; BC1530, Solarbio), urine protein (C035–2–1, Nanjing Jiancheng Bioengineering Institute, Nanjing, China), and plasma endotoxin (L00350, GenScript, Piscataway, NJ, USA).

### 2.5. Preparation of Medicated Mouse Serum Containing HJXJ

Healthy 8-week-old C57BL/6 mice (Shanghai Laboratory Animal Center, China) with similar weights were used in the experiment. The mice were administered 1 mL of 0.36 g/mL HJXJ orally via gavage twice a day, over 3 days. The mice were anesthetized using pentobarbital sodium (50 mg/kg, i.p.) 1 h after the final dose was administered, and blood samples were aseptically collected from the abdominal aorta under cooled conditions. Mice in the control group were administered an equivalent volume of physiological saline. The HJXJ-containing blood samples were then centrifuged at 2000 × g for 20 min to separate blood cells from plasma. The obtained serum was heat-inactivated at 56°C and subsequently filtered through a 0.22-*μ*m filter to ensure that the samples were sterile. The serum samples were aliquoted and stored at −70°C.

### 2.6. Cell Culture and Treatment

Mouse renal podocyte MPC5 cells (SNL-566), obtained from the American Type Culture Collection (ATCC; Manassas, VA, USA), were cultured in Dulbecco modified Eagle medium (DMEM; 30–2002, ATCC) supplemented with 10% fetal bovine serum (FBS; 30–2020, ATCC) and different concentrations of PS (0.1, 0.3, and 1 mM) for 48 h. MPC5 cells treated with 1 mM PS were cultured in DMEM supplemented with 10% FBS (control) or 8% FBS and 2% mouse-medicated serum containing HJXJ, or 5% FBS and 5% mouse-medicated serum containing HJXJ or 10% mouse-medicated serum containing HJXJ.

### 2.7. Gene Overexpression

The coding sequence of Nlrp3 was ligated into the pLVX-Puro plasmid (VT1465, YouBia, Hunan, China). Next, 293T cells (CC-Y1010, Ek-Bioscience, Shanghai, China) were transfected with pLVX-Puro-Nlrp3 for 4–6 h using Lipofectamine 2000 (11668019, Invitrogen, Millipore, USA). Subsequently, viruses were collected 48 h posttransfection and used to infect MPC5 cells. Blank pLVX-Puro (Vector) was used as the negative control.

### 2.8. ROS Detection

The DCFH-DA probe (MFCD00128955, BIOFOUNT, Beijing, China) was used to assess ROS level in MPC5 cells. Briefly, the DCFH-DA probe was added to the cell medium at a final concentration of 10 *μ*M. After incubation for 20 min at 4°C in the dark, fluorescence was detected by conducting flow cytometry assays at excitation/emission wavelengths of 485/530 nm.

### 2.9. Analysis of Pyroptosis Levels

The cells were treated with FLICA 660-YVAD-FMK (ab270784, Abcam, Cambridge, UK), and propidium iodide (PI) was used to mark the cells with membrane pores. The cells were exposed to 60× FLICA 660 and incubated for 1 h at 37°C in the dark. After incubation, the cells were washed thrice with 1× cell washing buffer and stained with 3 *μ*M PI for 15 min at 25°C. The CytoFLEX flow cytometry cytometer (Beckman, USA) was used to detect the percentage of thermophilic cells. The output images of pyroptosis experiments included four regions, wherein regions with active caspase-1^+^PI^+^ indicated the presence of pyroptotic cells.

### 2.10. Enzyme-Linked Immunosorbent Assay (ELISA)

Following the manufacturer's instructions, ELISA kits (Shanghai Jining Shiye Co., Ltd, Shanghai, China) were used to assess the concentrations of interleukin (IL)-1*β* (JN16939), IL-18 (JN17182), tumor necrosis factor-*α* (TNF-*α*) (JN17113), and IL-6 (JN16894). Additionally, a microplate reader (1510, Thermo Fisher, USA) was used to detect the OD value at 450 nm.

### 2.11. Quantitative Real-Time PCR

Cells were collected, centrifuged, and washed. Then, total RNA was extracted from these cells on ice using TRIzol (R0016, Beyotime, Shanghai, China). Thereafter, 2 *μ*g of RNA was reverse-transcribed into cDNA using the SuperScript™ II Reverse Transcriptase (Invitrogen). The reaction mixture for the SYBR Green Master Mix (ABI, Foster City, CA, USA) was prepared following the manufacturer's instructions, and qPCR was conducted on the Light Cycler® 96 PCR System (Roche, Basel, Switzerland). The qPCR thermal cycling conditions comprised an initial denaturation step at 95°C for 5 min, followed by 30 cycles of denaturation at 95°C for 30 s, annealing at 60°C for 30 s, extension at 72°C for 30 s, and a final extension at 72°C for 5 min. Each sample was run in triplicate at minimum. The relative mRNA expression level of Nlrp3 was calculated using the 2^−ΔΔCt^ method, with GAPDH serving as the internal reference gene. The primer sequences used were as follows: Nlrp3-F 5′-ACCTCCAAGACCACTACGG-3′, Nlrp3-R 5′-CAGCCAGTGAACAGAGCC-3′, GAPDH-F 5′-CCTTCCGTGTTCCTACCC-3′, and GAPDH-R 5′-CAACCTGGTCCTCAGTGTAG-3′.

### 2.12. Western Blotting

Protein extracts from mouse kidney tissue or the culture were prepared in radioimmunoprecipitation assay (RIPA) lysis buffer. Protein concentrations were determined by conducting the BCA assay (P0009, Beyotime). After quantification, the proteins were separated by sodium dodecyl sulfate-polyacrylamide gel electrophoresis (SDS-PAGE), and they were then electroblotted onto polyvinylidene difluoride (PVDF) membranes. Thereafter, the membranes were blocked with 5% skim milk for 1 h and probed with specific primary antibodies at 4°C for 12 h. The antibodies used were specific for podocin (ab181143), nephrin (ab216341), NLRP3 (ab263899), gasdermin D-N-terminal (GSDMD-N) (ab215203), ZO-1 (ab216880), occludin (ab216327) (Abcam, Cambridge, UK), Caspase-1 p20 (sc-398715, Santa Cruz Biotechnology, California, USA), and glyceraldehyde-3-phosphate dehydrogenase (GAPDH, #5174, CST, USA) at 4°C for 12 h, followed by secondary antibodies for 2 h. An enhanced chemiluminescence system was used to detect protein expression.

### 2.13. Data Analysis

The western blot band intensity was calculated from images using ImageJ software, version 1.48v, provided by the National Institutes of Health (Bethesda, Maryland, USA). Statistical analysis was conducted using SPSS version 20.0 (SPSS Inc., Chicago, Illinois, USA). Differential analyses employed ANOVA methods to assess significant differences among different groups. Data were presented as the mean ± standard deviation (SD), based on a minimum of three independent samples. Differences among and between groups were considered to be statistically significant at *P* < 0.05.

## 3. Results

### 3.1. Effects of HJXJ on PS-Induced Kidney Injury in db/db Mice

**Figure 1:**
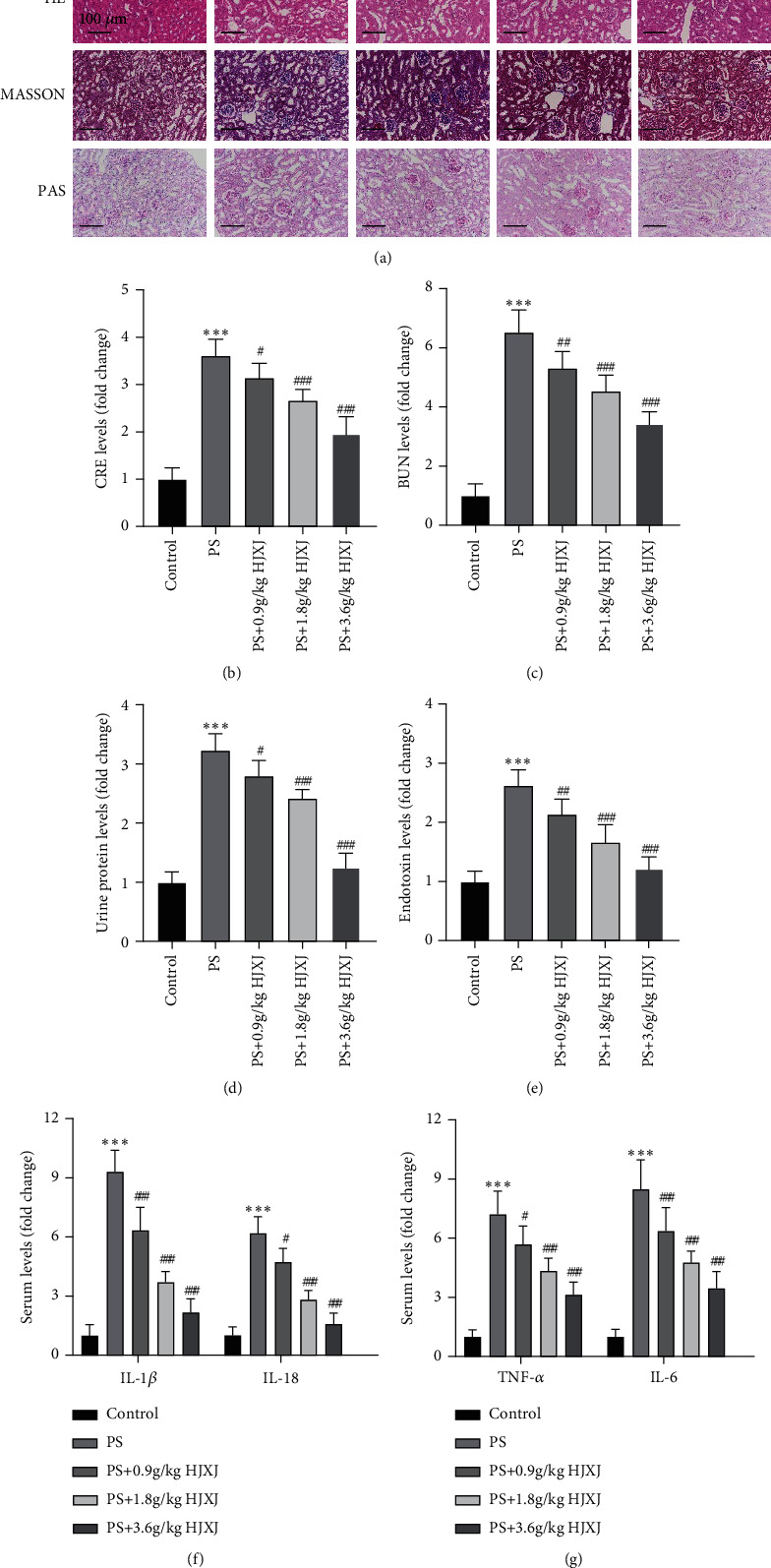
Huajuxiaoji formula (HJXJ) alleviates phenyl sulfate (PS)-induced kidney injury in db/db mice. (a) H&E, Masson trichrome (MASSON), and periodic acid–Schiff (PAS) staining of kidney tissues of db/m (control) and db/db mice with PS in the absence or presence of HJXJ. Scale bar: 100 *μ*m. (b–e) Specific assay kits detected the levels of (b) serum creatinine (CRE), (c) blood urea nitrogen (BUN), and in (d) urine samples and (e) plasma endotoxin. (f–g) Specific enzyme-linked immunosorbent assay (ELISA) kits detected serum levels of (f) IL-1*β* and IL-18 and (g) TNF-*α* and IL-6. Values are presented as mean ± SD. *n* = 6/group. ^∗∗∗^*P* < 0.001 versus. control. ^#^*P* < 0.05, ^##^*P* < 0.01, ^###^*P* < 0.001 versus PS.

To investigate the effects of HJXJ in DKD, kidney injury models were created using PS. As shown in Figure 1(a), the glomerular stroma and mesangial regions were significantly thickened in DKD mice, and the MASSON staining showed distinct fibers in the renal interstitium. However, interstitial fibrosis in mice significantly improved in each group after HJXJ treatment without any obvious atrophic lobulation. The results demonstrated that HJXJ offered beneficial effects against kidney injury in DKD.

Our results showed a significant increase in CRE, BUN, and urine protein in PS-treated mice compared with the control group, whereas HJXJ (0.9, 1.8, and 3.6 g/kg) significantly reversed these trends (Figures 1(b), 1(c), and 1(d), *P* < 0.05). Besides, our results showed higher endotoxin, IL-1*β*, IL-18, TNF-*α*, and IL-6 concentrations in the PS group compared with the control group. Interestingly, HJXJ (0.9, 1.8, and 3.6 g/kg) decreased these content levels compared with the PS group (Figures 1(e), 1(f), and 1(g), *P* < 0.05), indicating that HJXJ could significantly alleviate the injury and reduce the inflammatory response in the PS group mice dose-dependently (0.9, 1.8, and 3.6 g/kg) (Figure 1).

### 3.2. Effects of HJXJ on NLRP3-Mediated Pyroptosis in Mice With DKD

**Figure 2:**
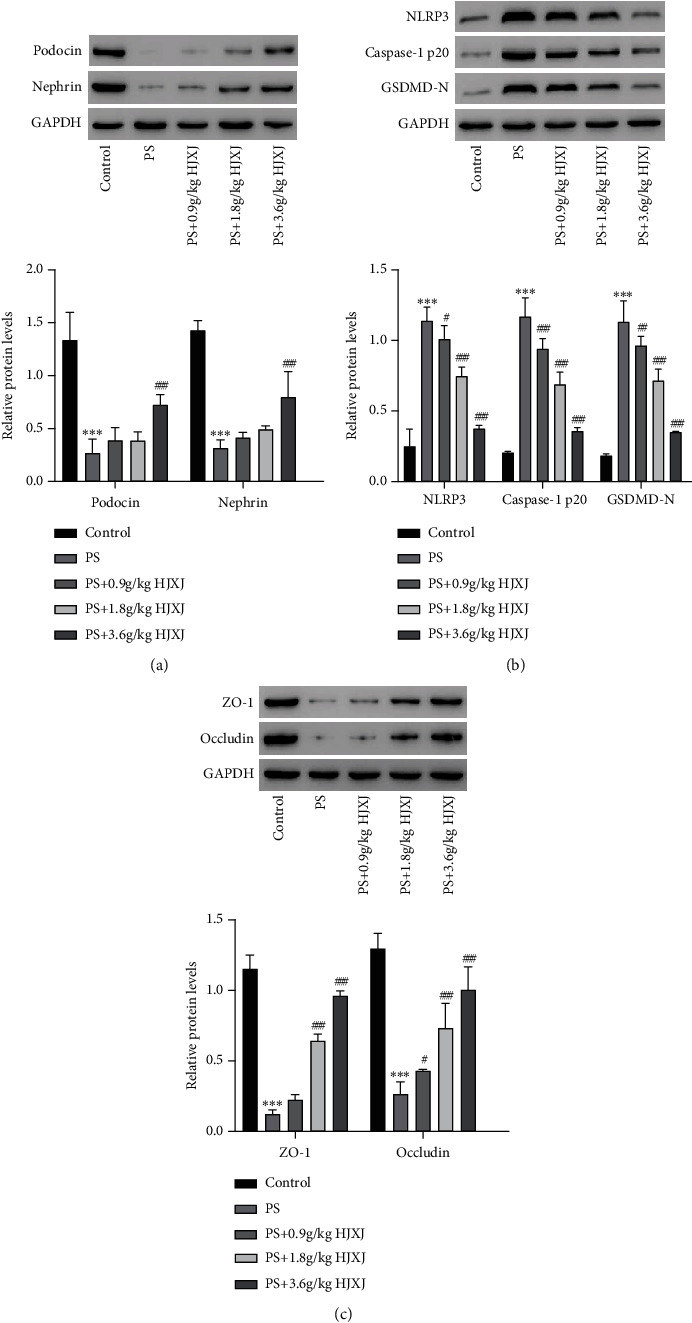
HJXJ reverses PS-induced related protein expressions in db/db mice. Western blotting is used to measure the expressions of (a) podocin, nephrin, (b) NLRP3, Caspase-1 p20, GSDMD-N in kidney tissues, and (c) ZO-1 and occludin in colon tissues of db/m (control) and db/db mice with PS in the absence or presence of HJXJ. Values are presented as mean ± SD. *n* = 6/group. ^∗∗∗^*P* < 0.001 versus control. ^#^*P* < 0.05, ^##^*P* < 0.01, ^###^*P* < 0.001 versus PS.

Podocin and nephrin are related to the pathogenesis of proteinuria [37]. Compared with the control group, the PS group showed significantly decreased protein expressions of podocin and nephrin (Figure 2(a), *P* < 0.001). However, these levels subsequently increased after HJXJ treatment (3.6 g/kg) (Figure 2(a), *P* < 0.001). Moreover, the protein levels related to pyroptosis, such as NLRP3, Caspase-1 p20, and GSDMD-N, were significantly increased due to PS treatment, whereas HJXJ (0.9, 1.8, and 3.6 g/kg) alleviated these effects (Figure 2(b), *P* < 0.05). Tight junction proteins, including occludin and ZO-1were measured to reflect the pyroptosis effect on cell permeability [38]. The results indicated that HJXJ (0.9, 1.8, and 3.6 g/kg) could increase the expression levels of occludin and ZO-1 compared with the PS group (Figure 2(c), *P* < 0.05). Our findings suggested that HJXJ alleviate DKD in mice through regulating the NLRP3-mediated pyroptosis pathway.

### 3.3. Effects of PS on NLRP3-Mediated Pyroptosis in MPC5 Cells

**Figure 3:**
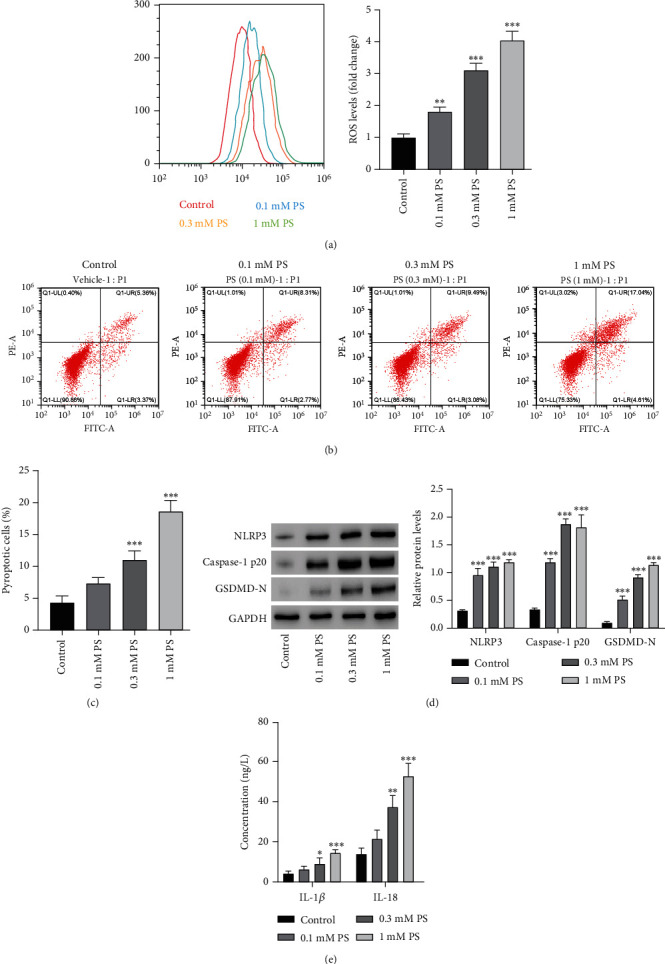
PS induces reactive oxygen species (ROS) production, pyroptosis, and NLRP3 inflammasome activation in MPC5 cells. MPC5 cells are treated with different PS concentrations. Flow cytometry is used to evaluate (a) ROS production and (b, c) pyroptosis level. Western blotting is used to measure (d) expressions of NLRP3, Caspase-1 p20, and GSDMD-N. ELISA kits are used to measure (e) release of IL-1*β* and IL-18. ^∗^*P* < 0.05, ^∗∗^*P* < 0.01, ^∗∗∗^*P* < 0.001 versus control.

To investigate the effects of PS on MPC5 cells, MPC5 cells were treated with various PS concentrations (0.1, 0.3, and 1 mM). Flow cytometry results showed that PS exposure caused a significant increase in ROS level (Figure 3(a), *P* < 0.05) and a higher number of cells positive for pyroptosis (Figures 3(b) and 3(c), *P* < 0.01). Western blotting results showed that PS (0.1, 0.3, and 1 mM) resulted in high expression levels of NLRP3, Caspase-1 p20, and GSDMD-N (Figure 3(d), *P* < 0.005), indicating that PS was associated with NLRP3 inflammasome activation. Moreover, IL-1*β* and IL-18 levels were increased after PS exposure (0.3 and 1 mM) (Figure 3(e), *P* < 0.05).

### 3.4. Effects of HJXJ on NLRP3-Mediated Pyroptosis in MPC5 Cells

**Figure 4:**
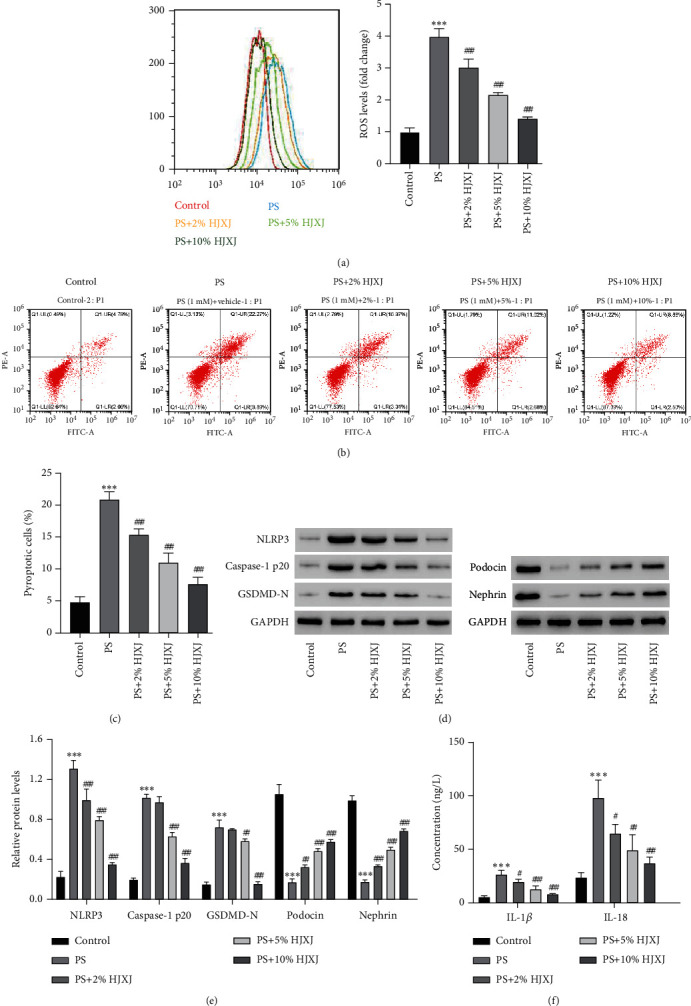
HJXJ alleviates PS-induced ROS production, pyroptosis, and NLRP3 inflammasome activation in MPC5 cells. MPC5 cells are treated with 1 mM PS in the absence or presence of different HJXJ concentrations. Flow cytometry was used to evaluate (a) ROS production and (b, c) pyroptosis. Western blotting is used to measure expressions of NLRP3, Caspase-1 p20, GSDMD-N, podocin, and nephrin (d, e). ELISA kits are used to measure (f) release of IL-1*β* and IL-18. ^∗∗∗^*P* < 0.001 versus control. ^#^*P* < 0.05, ^##^*P* < 0.01, ^###^*P* < 0.001 versus PS.

To evaluate the effects of HJXJ on pyroptosis in MPC5 cells, the cells were treated with varying HJXJ concentrations after exposing them to 1 mM PS. The results showed that HJXJ (2%, 5%, and 10% content) could reverse the increase in ROS induced by PS dose-dependently (Figure 4(a), *P* < 0.01). HJXJ (2%, 5%, and 10% content) also reduced pyroptotic cells caused by PS (Figures 4(b) and 4(c), *P* < 0.01). Western blotting results showed that PS caused significant upregulation of NLRP3, Caspase-1 p20, and GSDMD-N protein levels, which were inhibited by HJXJ dose-dependently (Figure 4(d), *P* < 0.01). Furthermore, HJXJ (2%, 5%, and 10% content) upregulated the levels of podocin and nephrin, which were inhibited by PS (Figures 4(d) and 4(e), *P* < 0.001), and decreased the content levels of IL-1*β* and IL-18 enhanced by PS (Figure 4(f), *P* < 0.05).

### 3.5. Effects of HJXJ on NLRP3 Overexpression-Induced Pyroptosis in MPC5 Cells

**Figure 5:**
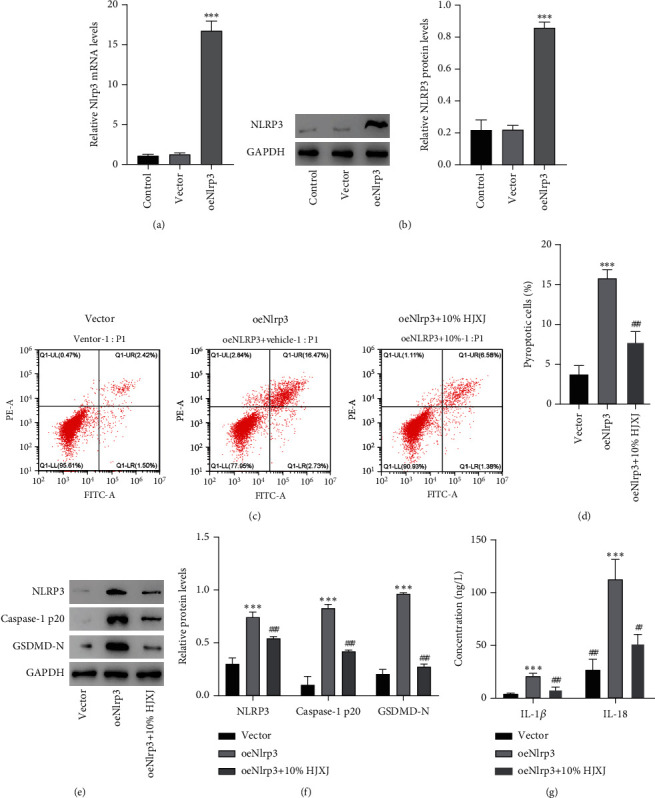
HJXJ alleviates Nlrp3 overexpression-induced pyroptosis and NLRP3 inflammasome activation in MPC5 cells. MPC5 cells are transduced with Nlrp3 expression vector or blank vector. (a) The NLRP3 mRNA expression is measured using quantitative real-time PCR, and (b) protein expression is measured using western blotting. MPC5 cells are pretreated with 10% HJXJ and transduced with Nlrp3 expression vector. Flow cytometry is used to evaluate (c, d) pyroptosis level. Western blotting is used to measure (e, f) expressions of NLRP3, Caspase-1 p20, and GSDMD-N. ELISA kits are used to measure (g) release of IL-1*β* and IL-18^.∗∗∗^*P* < 0.001 versus vector. ^#^*P* < 0.05, ^##^*P* < 0.01, ^###^*P* < 0.001 versus oeNlrp3.

To study the influence of HJXJ on NLRP3 and its involvement in DKD development, NLRP3 was overexpressed using a lentiviral vector to assess HJXJ's impact on PS-treated cells. As expected, NLRP3 overexpression enhanced the expression levels of NLRP3 mRNA and protein (Figures 5(a) and 5(b), *P* < 0.001), which showed successful transfection. Moreover, NLRP3 overexpression increased pyroptosis level in MPC5 cells compared with the vector group, whereas HJXJ (10% content) significantly reversed this trend (Figures 5(c) and 5(d), *P* < 0.001). Western blotting results indicated that NLRP3, Caspase-1 p20, and GSDMD-N significantly increased in the oenlrp3 group, which HJXJ (10% content) could inhibit (Figures 5(e) and 5(f), *P* < 0.001). In terms of the inflammatory response, the results showed that the NLRP3 overexpression group had higher IL-1*β* and IL-18 levels compared with the vector group; however, HJXJ could decrease this trend (10% content) (Figure 5(g), *P* < 0.01). These results suggested that NLRP3 was an important target of HJXJ for inhibiting pyroptosis.

## 4. Discussion

Maintaining glycemic control and managing blood pressure (through inhibition of the renin-angiotensin-aldosterone system) remain fundamental in managing DKD, with added emphasis on nephroprotective medication choices that target glycemic control, hypertension, lipid abnormalities, proteinuria, uric acid management, addressing edema, anemia, and other associated conditions [39]. The use of Chinese herbs for treating DKD has been widespread in China. Our findings supported the preventative effects of HJXJ on the progression of DKD. HJXJ could suppress the inflammatory response and pyroptosis by inhibiting the activation of the NLRP3 inflammasome and reducing ROS production. This study indicated a significant strategy for alleviating DKD by targeting the inflammatory response and pyroptosis pathways involving NLRP3.

PS is a metabolite derived from the intestinal flora. Kikuchi et al. have reported that PS serves as an early diagnostic marker and a modifiable etiology, making it a potential therapeutic target for DKD [36]. PS plays a role in cellular injury, specifically targeting podocyte integrity and function [40]. *In vivo* experiments demonstrated that the DKD mice exhibited elevated levels of CRE, BUN, urine protein, and endotoxin, which are important indicators for evaluating kidney function [41, 42]. After treating the mice with HJXJ, their behavior and symptoms saw a significant improvement overall, which is consistent with previous studies [31]. *Astragalus mongholicus* is a key active ingredient found in HJXJ. It is noteworthy that *Astragalus mongholicus* may have a significant meaning in HJXJ, as it has the ability to regulate gut microbiota and blood glucose [43–45], as well as prevent fibrosis [46]. Additional research can be conducted to evaluate if/how HJXJ impacts PS in patients with DKD.

A previous study linked sterile inflammation to DKD, and NLRP3 inflammasome activation was considered a contributing factor in the development of DKD [47]. Highly expressed NLRP3 inflammasome has been detected in the renal tubules of diabetic patients with tubular interstitial injury [48]. Our results demonstrated that HJXJ effectively inhibited the level of NLRP3 in DKD models, thus affecting the formation and activation of NLRP3 inflammasome. The limited expression of NLRP3 itself accounts for the requirement of priming of the NLRP3 inflammasome [49]. HJXJ-induced downregulation of NLRP3 may be involved in the decrease of NF-*κ*B activity [50–54]. In fact, we found that ROS levels were reduced in HJXJ-treated cells. ROS signaling serves as an upstream event that regulates NLRP3 expression, promoting the activation of inflammasome [55, 56]. ROS-mediated pyroptosis is also associated with thioredoxin-interacting protein (TXNIP) [54], nuclear factor E2-related factor 2 (Nrf2) [57], and AMP-activated protein kinase (AMPK) [58]. It is reported that *Centella asiatica* contributes to the clearance of ROS [59]. Some herbal ingredients, such as salidroside (NF-*κ*B/NLRP3 axis) [60], lonicerin (mitochondrial autophagy) [61], andrographolide (triggering mitochondrial dysfunction) [62], and celastrol (K63 deubiquitination) [63], have been found to play a role in lung injury, ulcerative colitis, renal tubulointersticial injury, and liver damage through targeting the NLRP3 inflammasome. Additionally, ginsenosides have been reported to have a strong affinity for NLRP3 and can directly bind to the NLRP3 amino acid site [64]. Parthenolide is considered a direct inhibitor of inflammasome due to its ability to alkylate cysteine residues in the Caspase-1 and NLRP3 ATPase domains [65, 66]. Network pharmacological prediction helps in identifying the binding site and strength of drug-target interactions. Further clarification is required to determine whether the active components of HJXJ can directly interact with NLRP3.

NLRP3 has been shown to mediate renal damage in DKD models by regulating podocyte pyroptosis [67, 68]. Mechanistically, pyroptosis is executed by caspase-dependent GSDMD. GSDMD mediates pyroptosis levels in renal diseases and inhibits GSDMD levels to alleviate renal injury [69, 70]. Inflammatory caspases cleave GSDMD into N- and C-terminal regions and then GSDMD N-terminal domains oligomerize in cell membranes to form membrane pores, leading to the release of inflammatory mediators IL-18 and IL-1*β*, which induce an inflammatory cascade amplification reaction that eventually results in inflammatory cell death [71]. In our study, it was observed that inflammatory cytokines accumulated in the DKD mice kidneys, indicating an inflammatory state. And the *in vitro* and *in vivo* results confirmed that HJXJ inhibited the NLRP3/Caspase-1/GSDMD/IL-1*β* signaling cascade. Additionally, there is substantial evidence suggesting that various specific forms of regulatory necrosis contribute to different models of nephrotoxicity, and necrotic inflammation or immunogenic cell death serves to worsen the extent of tissue damage [14]. TNF-*α* and IL-8 can induce NETosis, which leads to cytotoxic effects on endothelial cells and mesangial cells [72]. Not only does HJXJ reduce the loss of renal units by inhibiting pyroptosis, but it can also minimize additional tissue damage caused by the inflammatory environment. Interestingly, components of HJXJ, such as *Astragalus mongolicus*, *Rhizoma coptis chinensis*, and *Centella asiatica*, have shown anti-inflammatory effects and are involved in regulating the inflammasome pathway [51, 73, 74].

The stable attachment and function of podocytes to the glomerular basement membrane depend on a group of proteins related to podocytes. Mature IL-1*β* has the potential to impair the structural integrity and function of podocyte proteins by affecting the production of podocyte proteins, especially renin [75]. Both nephrin and podocin are key components of the slit diaphragm in the glomerular filtration barrier. Nephrin affects the permeability and matrix deposition of the glomerular basement membrane by mediating the interaction between epithelial cells and matrix; thus, the integrity of the renal filtration membrane structure and barrier function is affected [76]. Podocin can interact with both nephrin and CD2AP through its carboxyl terminus to participate in various signaling events at the slit diaphragms [77]. Downregulating the protein expression level of nephrin and podocin leads to proteinuria, and a greater degree of podocyte injury results in more serious structural damage of the hiatal membrane between podocytes [78]. Our study showed that HJXJ could promote the expression of nephrin and podocin protein, suggesting that HJXJ might alleviate podocyte barrier damage caused by PS. Notably, Huwiler et al. found that the mRNA and protein levels of nephrin increased after exposure to IL-1*β* and TNF-*α* in human embryonic kidney cells and human podocellular primary cultures of A293 [79]. The damage to the podocyte barrier may be a consequence of a sustained accumulation of inflammation.

## 5. Conclusion

Taken together, our study highlighted the significant roles of NLRP3-mediated inflammasome activation and pyroptosis in the prevention of DKD progression in HJXJ. These findings shed new light on the mechanisms of NLRP3 inflammasome and pyroptosis in treating DKD.

## Data Availability

The data used to support the findings of this study are available from the corresponding author upon request.
